# Cytokine autoantibodies are stable throughout the haematopoietic stem cell transplantation course and are associated with distinct biomarker and blood cell profiles

**DOI:** 10.1038/s41598-021-01952-6

**Published:** 2021-12-14

**Authors:** Jakob Hjorth von Stemann, Lars Klingen Gjærde, Eva Kannik Haastrup, Lia Minculescu, Patrick Terrence Brooks, Henrik Sengeløv, Morten Bagge Hansen, Sisse Rye Ostrowski

**Affiliations:** 1 Department of Clinical Immunology, Rigshospitalet, University of Copenhagen, Copenhagen, Denmark; 2 Department of Haematology, Rigshospitalet, University of Copenhagen, Copenhagen, Denmark

**Keywords:** Immunological disorders, Autoimmunity, Cytokines, Immunological disorders, Transplant immunology

## Abstract

Cytokine-specific autoantibodies (c-aAbs) represent an emerging field in endogenous immunodeficiencies, and the immunomodulatory potential of c-aAbs is now well documented. Here, we investigated the hypothesis that c-aAbs affects inflammatory, immunoregulatory and injury-related processes and hence the clinical outcome of haematopoietic stem cell transplantation (HSCT). C-aAbs against IL-1α, IL-6, IL-10, IFNα, IFNγ and GM-CSF were measured in 131 HSCT recipients before and after (days + 7, + 14, + 28) HSCT and tested for associations with 33 different plasma biomarkers, leukocyte subsets, platelets and clinical outcomes, including engraftment, GvHD and infections. We found that c-aAb levels were stable over the course of HSCT, including at high titres, with few individuals seeming to acquire high-titre levels of c-aAbs. Both patients with stable and those with acquired high-titre c-aAb levels displayed significant differences in biomarker concentrations and blood cell counts pre-HSCT and at day 28, and the trajectories of these variables varied over the course of HSCT. No clinical outcomes were associated with high-titre c-aAbs. In this first study of c-aAbs in HSCT patients, we demonstrated that high-titre levels of c-aAb may both persist and emerge in patients over the course of HSCT and may be associated with altered immune biomarkers and cell profiles.

## Introduction

Cytokine-specific autoantibodies (c-aAbs) represent a relatively new field in the study of immune dysfunction, with the International Union of Immunological Societies reporting c-aAbs as a phenocopy of primary immune deficiency in 2019^1^. C-aAbs against a wide range of cytokines have been reported, and c-aAbs are widely detectable even in a healthy population^2^. This includes c-aAbs at high titre levels, which appear to be stable for several years^3^. High-titre c-aAbs are associated with increased age and biological sex, but their exact aetiology is unknown. The cytokine-inhibitory effects of high-titre c-aAbs have been verified in vitro^3^, and recently, we found associations between c-aAbs against pro- and anti-inflammatory cytokines and an altered infection risk in otherwise healthy blood donors^4^. High-titre c-aAbs against interleukin (IL)-6 have also been linked to low levels of IL-6 and CRP, even in healthy blood donors and have been associated with several staphylococcus infection cases^2,5–7^. Recently, c-aAbs against IFNγ has been associated with mycobacterial infections^8,9^, and high-titre IFNα c-aAbs have been found to correlate with a poor disease course in COVID-19^10,11^.

The production of endogenous IFNα c-aAb has been linked to mutations of the AIRE and RAG genes^12–15^, and c-aAb in general have been implicated in many pathologies comparable to established cytokine-related primary immune deficiencies^16^. Antibodies against granulocyte–macrophage colony stimulating factor (GM-CSF) in particular have been extensively characterized and have been designated a diagnostic factor for the respiratory disease pulmonary alveolar proteinosis, which arises from macrophage dysfunction caused by GM-CSF suppression^17,18^. GM-CSF-specific c-aAbs are also associated with inflammatory diseases, including Crohn’s disease, ulcerative colitis and endometriosis^19,20^, as well as cryptococcal infections^16,21–23^. However, the presence of c-aAbs against the proinflammatory cytokine IL-1α have also been associated with improved prognosis in autoinflammatory diseases such as arthritis, and IFNα c-aAbs have been inversely correlated with type 1 diabetes^15,24^, suggesting that their ultimate effect is variable and context-specific.

In this study, we investigated c-aAbs in patients undergoing haematopoietic stem cell transplantation (HSCT). HSCT represents a unique immunologic situation with a switch in the origin of the immune system, and based on the crucial role of the immune system—and cytokines in particular—for obtaining successful engraftment and avoiding complications such as graft vs host disease (GvHD) and infection, we also found this a highly relevant setting to further reveal the immunomodulatory impact of c-aAbs. We obtained access to a longitudinal cohort of well-characterized HSCT patients with a concomitantly high number of biomarkers measured in parallel and hypothesized that functional cytokine inhibition by high-titre levels of c-aAb would influence the levels and course of immunological biomarkers and cells in HSCT and hereby potentially also clinical outcomes. This could ultimately help explain part of the differences in clinical outcomes still observed among HSCT patients. For a description of patient and transplant characteristics, see (Table 1).

**Table 1:** Patient and transplant characteristics.

Age at HSCT, years	49.6 (35.5; 57.9)^a^
Male sex, n (%)	71 (54%)
Disease	Malignant (AML, ALL, CML, HL, MDS, MM/PCD, NHL, Other leukaemia): 91% Non-malignant (other): 9%
Karnofsky score	90 (49%)/100 (32%)/ < = 80 (19%)
Smoking status at HSCT	Active/Former Smoker (42%)/Never Smoked (44%)/Unknown (14%)
Body mass index, kg/m^2^	24.4 (22.1; 28.6)^a^
CD34 cell count	420 (192; 608)^a^
Donor age, years	33 (+ 27)
Donor match^b^	Matched unrelated donor (66%), matched related donor (24%), mismatched unrelated donor (7%), haploidentical donor (4%)
Stem cell source	Peripheral blood (65%), bone marrow (34%), umbilical cord blood (2%)
Conditioning regimen	Fludarabine + Treosulfan (49%), cyclophosphamide + TBI (32%), other (19%)
Anti-thymocyte globulin	32 (24%)

^a^Median (1st quartile; 3rd quartile).

^b^Matched = 9/10 or 10/10 HLA allele match, mismatched = one or more HLA antigen mismatches.

## Results

### Cytokine autoantibodies

We detected cytokine-specific autoantibodies (c-aAbs) in all patients, and the distributions and thresholds for high-titre c-aAbs are displayed in Table 2. The highest c-aAb levels observed were against IL-1α and IL-6 (95th percentile threshold at almost 10 × median), with IL-10 c-aAbs having the lowest levels of expression (95th percentile threshold at approximately 5 × median, Table 2). The prevalence and level of c-aAbs compared to each other in HSCT patients appeared similar to the pattern of c-aAbs observed in healthy individuals (Fig. S1), though with relatively higher levels of IL-1α-, IL-6- and IFNγ-aAbs in patients compared to healthy individuals. All c-aAbs seemed comparable between healthy donors and patients in terms of the 99th percentile cut-off for c-aAb MFI.

**Table 2:** c-aAb distribution and stability.

	GM-CSF c-aAbs		IL-10 c-aAbs		IFNα c-aAbs		IFNγ c-aAbs		IL-1α c-aAbs		IL-6 c-aAbs	
**Distribution (MFI)**												
Median (1st quartile; 3rd quartile)*	160 (98; 402)		111 (69; 176)		142 (93; 227)		462 (275; 774)		262 (171; 499)		293 (172; 572)	
High-titre threshold**	1758		582		605		2,567		2,499		2,431	
Stability (CV%)***												
Non-high titre**	18.5 (11.3; 36.3)	*P* = 0.08****	16.8 (9.5; 25.0)	*P* = 0.50****	16.2 (10.8; 23.2)	*P* = 0.48****	17.5 (10.7; 31.9)	*P* = 0.50****	19.6 (11.2; 31.4)	*P* = 0.41****	17.6 (10.1; 26.4)	*P* = 0.27****
High titre **	8.9 (7.7; 11.4)		10.1 (8.1; 35.9)		12.2 (12.1; 18.5)		11.5 (10.5; 12.9)		18.2 (11.3; 20.9)		21.2 (16.9; 25.1)	

*Based on average c-aAb MFI/person.

**High titre defined as > 95th percentile MFI cut-off for average c-aAb/person.

***Median (1st quartile; 3rd quartile) for CV% of c-aAb/person.

****Mann–Whitney *U*-test, CV% comparison between c-aAb high/non-high titre groups, *P*-value reported.

All c-aAbs tended to correlate positively with each other in terms of MFI signals both pre-HSCT and at day + 28, with IL-1α and IL-10, IL-1α and IFNα, and IL-6 and IFNγ c-aAbs showing positive correlations at the high titre levels, with two shared individuals each (Table S1).

### Cytokine autoantibody stability during HSCT

**Figure 1:**
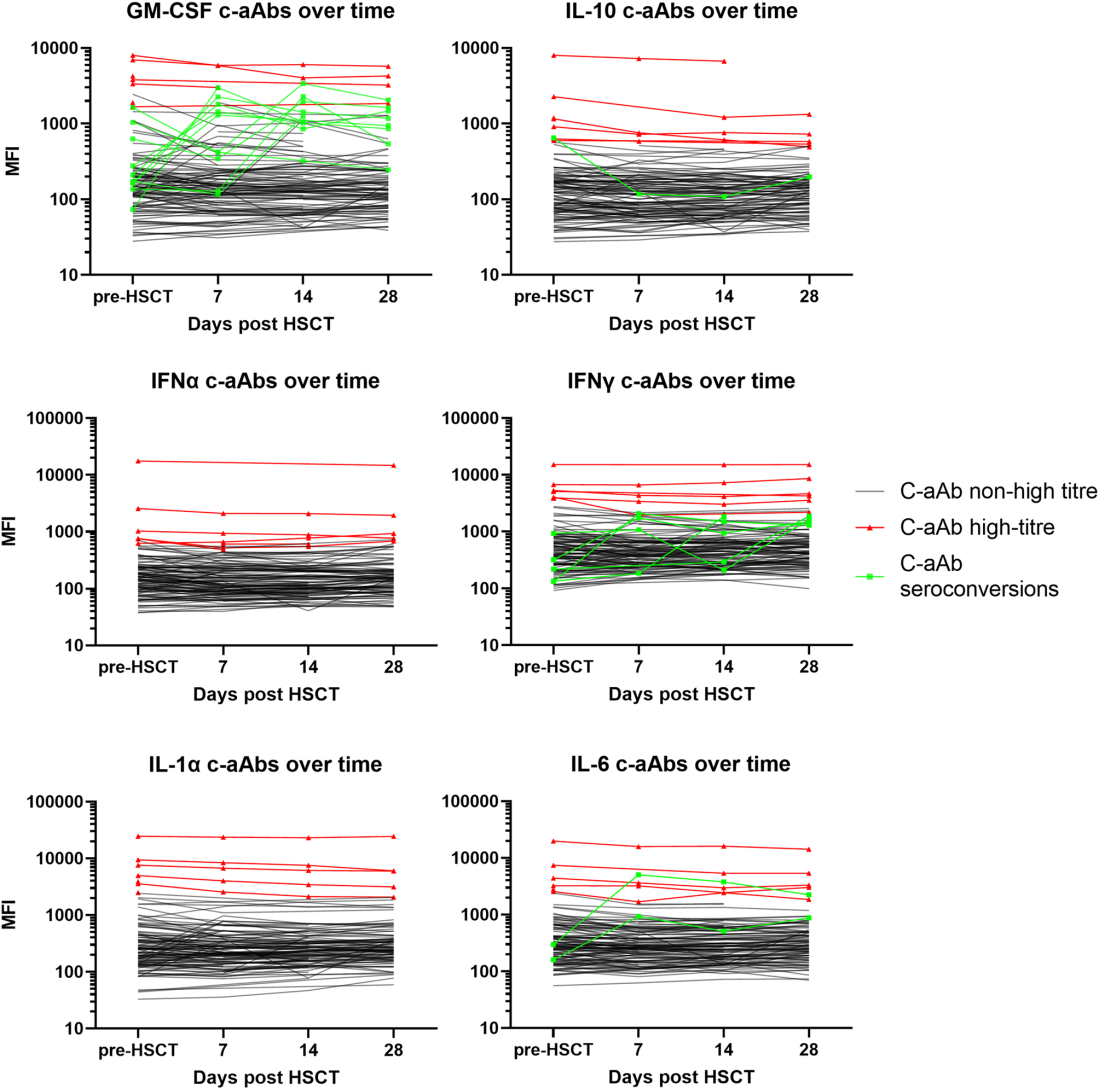
Cytokine autoantibody levels over time. C-aAb levels for all patients (n = 131) during HSCT (each line represents one patient). Patients who were classified as having high-titre c-aAbs (≥ 95th percentile for a person’s average MFI score) are coloured red with triangle icons. Patients with seroconversions, defined as maximum observed c-aAb MFI > 90th percentile and a (maximum c-aAb MFI/minimum c-aAb MFI) factor difference of ≥ 5, are coloured green with square icons.

The c-aAb signals were stable over time (Fig. 1) across the sample dates from days − 71 to + 37 (the extremes of our samples pre-HSCT and around day 28), with median CV%s below 20% for all non-high-titre c-aAbs, and median CV%s for high-titre c-aAbs being even lower (except c-aAbs against IL-6, with a CV% of 21%). The CV% between high-titre and non-high-titre c-aAbs were comparable (Table 2).

A few patients experienced drastically altered c-aAb levels during HSCT. For the GM-CSF c-aAbs, 9 patients “seroconverted” with increasing c-aAb levels changing from MFI signal values more than a factor five higher to levels above the 90th percentile (Fig. 1). One patient had a more than fivefold reduction in MFI signal (Table 3). Furthermore, four patients “seroconverted” with fivefold higher IFNγ c-aAb levels to levels above the 90th percentile (Fig. 1).

**Table 3:** “Seroconverting”* cases with increasing GM-CSF and IFNγ c-aAbs over the duration of HSCT vs cells and biomarkers.

Biomarker/cell	Cohort median (IQR)	Seroconverting median (IQR)	*P*-value**
**Increasing GM-CSF c-aAb (n = 9)**			
*Pre-HSCT associations*			
IL-10 (pg/ml)	1.1 (0.7; 1.8)	1.6 (1.2; 2.6)	0.043
Reg3A (pg/ml)	7147 (5174; 10,749)	10,654 (7812; 13,159)	0.026
**Increasing IFNγ c-aAb (n = 4)**			
*Pre-HSCT associations*			
IL-2Ra (pg/ml)	2759 (2080; 3515)	3660 (3615; 4576)	0.011
sTNFRI (pg/ml)	1793 (1482; 2291)	2363 (2190; 2721)	0. 037
*Post-HSCT (day 28) associations*			
IL-8 (pg/ml)	12.7 (7.9; 22.3)	25.8 (19.8; 57.8)	0.024
Platelets (10E9 cells/L)	97 (38; 166)	12 (11; 25)	0.002

*“Seroconversion” defined as having a maximum observed c-aAb MFI > 90th percentile and a (maximum c-aAb MFI/minimum c-aAb MFI) factor difference of ≥ 5.

**Mann–Whitney *U*-tests, concentration comparison between c-aAb-increasing seroconverting individuals and non-seroconverting individuals pre or post HSCT, *P*-value reported.

The “seroconverting” individuals who experienced an increase in GM-CSF c-aAbs over the course of HSCT had significantly higher concentrations of IL-10 and Reg3A pre-HSCT than nonconverting patients (Table 3). Patients with increasing IFNγ c-aAbs over time had significantly higher concentrations of IL-2Ra and sTNFRI pre-HSCT and higher concentrations of IL-8 at day 28, as well as reduced platelet counts at day 28 (Table 3).

### Associations between pre-HSCT cytokine autoantibodies, demography and disease characteristics

Pre-HSCT high-titre IL-10 c-aAbs (i.e., as a binary variable) were associated with younger age (*P* = 0.024), and pre-HSCT high-titre IFNγ c-aAbs were associated with lower BMI (*P* = 0.032). Furthermore, several c-aAbs were associated with disease type; IL-1α c-aAbs levels were higher in patients with nonmalignant disease (*P* = 0.050), and the proportion of nonmalignant patients with high-titre levels of GM-CSF and IL-10 c-aAbs was elevated (86% of high-titre individuals, *P* = 0.032 in both cases), whereas all individuals with high-titre IFNγ c-aAbs had malignant disease (*P* = 0.013). No associations between c-aAbs and conditioning regimen or donor characteristics were observed.

When investigating the influence of average high-titre c-aAbs on clinical outcome, no significant associations were observed.

### Pre-HSCT cytokine antibodies and biomarker levels and blood cell counts

We investigated the association between the investigated c-aAbs and available biomarkers and cell counts at the pre-HSCT time point.

High-titre pre-HSCT levels of GM-CSF c-aAbs were associated with higher monocyte counts, and IL-10 c-aAbs were associated with lower IL-7 concentrations (Table S2).

High-titre IFNα c-aAbs were associated with higher IL-6, IL-17A, TNFα and ST2 concentrations, and IFNγ c-aAbs were associated with lower concentrations of syndecan-1.

High-titre pre-HSCT IL-1α c-aAbs were associated with higher concentrations of ST2 and lymphocyte counts.

High-titre IL-6 c-aAbs were associated with lower thrombomodulin concentrations.

We also found biomarker concentration and cell count differences from the cohort when investigating individual cases with the highest titre levels of an individual c-aAb, as well as one case with four high-titre c-aAbs (see supplementary Table S3).

### Cytokine autoantibodies are associated with blood cell and biomarker trajectories post-HSCT

**Figure 2:**
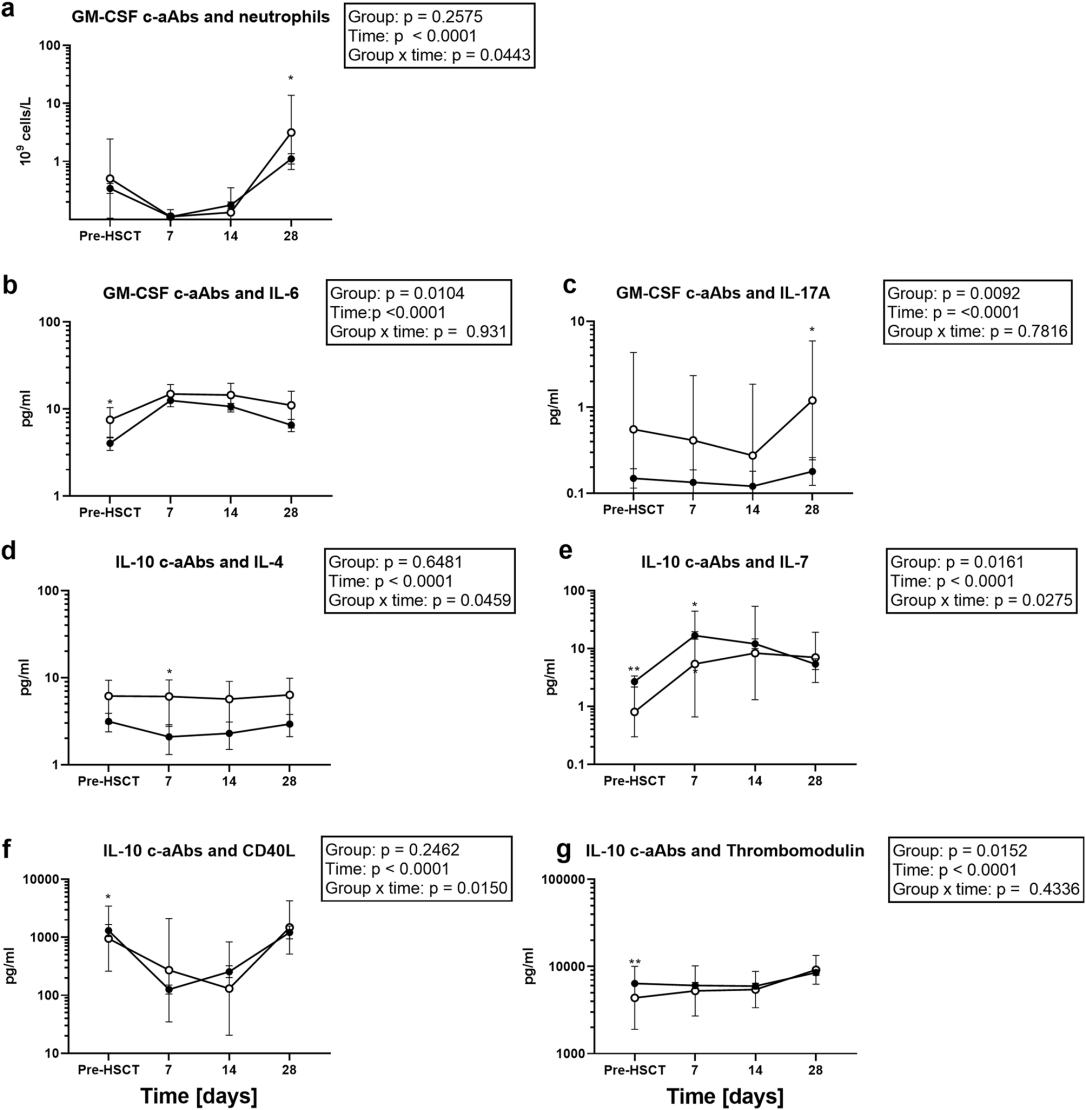
GM-CSF and IL-10 c-aAbs as predictors for cell and biomarker trajectories pre- and post-HSCT. Results from linear repeated mixed measures models of all patients (n = 131) with biomarker concentrations and cell counts as dependent variables and time and high-titre c-aAbs as independent variables. Analyses performed in Stata (StataCorp. 2019. *Stata Statistical Software: Release 16*. College Station, TX: StataCorp LLC). Filled circles represent individuals with nonhigh titres of c-aAb, and clear circles represent individuals with high titres of c-aAb. Data in (**a**, **c**), and (**e**–**g**) are geometric means with 95% confidence intervals in the case of log-transformed variables. Data in (**b**) and (**d**) are estimated mixed model regression coefficients with 95% confidence intervals in the case of untransformed variables. *P*-values are displayed for group (c-aAb high-titre or not), time and group × time effects as assessed by likelihood ratio post hoc tests. The effect of high c-aAb levels on biomarker levels compared to nonhigh c-aAb levels on a date-by-date basis was assessed by testing for a time/c-aAb interaction term as an outcome predictor; here * denotes *P*-value < 0.05 and ** denotes *P*-value < 0.01. Only results for cells or biomarkers with a significant group or group × time effect and with normally distributed residuals are shown. The results with a significant group × time effect with oscillating signals at the day 7 and 14 time points were excluded as biologically irrelevant.

High-titre GM-CSF-specific c-aAbs were the only c-aAb that influenced blood cell counts in the mixed model analysis, as it was associated with increasing neutrophil counts over time and higher day 28 neutrophil counts (Fig. 2a).

High-titre GM-CSF c-aAbs over the HSCT course were also associated with higher IL-6 and IL- 17A in general, and higher IL-6 and IL-17A concentrations at days 0 and 28, respectively (Fig. 2b, c).

High-titre IL-10 c-aAbs were associated with different trajectories for IL-4, IL-7 and CD40L over time. High-titre IL-10 c-aAb were also associated with lower concentrations of thrombomodulin in general, as well as pre-HSCT (Fig. 2c–g).

## Discussion

In this study, we demonstrated the presence of c-aAbs in patients undergoing HSCT. C-aAbs were present prior to transplant, some in high titres, and they remained stable the first 4 weeks after transplantation. The relative levels of c-aAbs in comparison to each other, as well as the overall distributions of the investigated c-aAb MFIs in the patients, were largely similar to those observed in a healthy cohort^2^, particularly at the 99th percentile level of high-titres. Although most c-aAb levels were weakly but universally intercorrelated both pre-HSCT and at day 28, overlap at high-titre levels was limited, and none of the cases with the highest observed titres of c-aAb overlapped, in accordance with prior studies^2^.

Beyond verifying their presence in a novel cohort, our study was a unique chance to investigate the pathophysiology of c-aAbs by monitoring their levels in the face of a complete replacement of an individual’s immune system. In a previous study, we found c-aAb titres to be relatively stable over years even at high titres in healthy individuals^3^. Likewise, in this study, with both high and nonhigh titres of pre-HSCT c-aAbs, these signals were remarkably stable after transplantation, in a time frame crossing the threshold of expected IgG half-life (3–4 weeks). The fact that some patients had specific high-titre c-aAbs pre- and post-HSCT may suggest that the autoantibodies originate from a retained and enduring population of c-aAb-producing plasma cells that survived the conditioning regimen. It is also possible that these persistently high-titre individuals have some intrinsic factor(s) that provoke c-aAb generation, separate from the immune progenitor cells themselves, but it must be noted that the follow-up period in this study, ends before full reconstitution of new B cells can be expected. The stability of high-titre c-aAb in this context however indicates that any c-aAb-mediated immunomodulatory effects might have potential long-term influences on outcomes in a manner comparable to cases of retained A or B glycoprotein antibody-producing plasma cells in blood type 0 transplant patients^25,26^. Further studies are thus highly warranted to investigate this further.

A few patients seemed to experience “seroconversions” of c-aAbs; that is, drastically changed c-aAb levels after initiation of HSCT, particularly GM-CSF and IFNγ c-aAbs. Several differences in biomarker concentrations and blood cell counts were seen pre-HSCT and at day 28 in both “seroconverters” and stable high-titer patients compared to “non-seroconverters” and non-high-titer patients respectively, suggesting that there may be intrinsic differences and, potentially, variable HSCT outcomes for such patients. This change in c-aAb level may be caused by a number of factors, including transplant donors having high-titre c-aAbs, as the occurrence of high titres above those seen in the patients in this cohort can be found in 1% of a healthy population^2^. The change may also arise from blood transfusions, since the time frame of the study (28 days post HSCT) represents a period with a high rate of transfusions for the patients, and blood donors may, similar to transplant donors, provide high-titre c-aAbs. It should however be emphasized that high-titre c-aAb are mainly expected to be transferred by plasma, and most patients received no or very little plasma in comparison to RBC and platelets (we use buffy coat platelets suspended in platelet additive solution i.e. only limited amounts of plasma are present in our platelet components). It is also possible that the transplant triggers loss of immunologic tolerance for the cytokines in question—GM-CSF specifically is a highly immunogenic molecule to the point where it has been phased out of many clinical treatments^27^. The aetiology of these “seroconversions”, however, remains unknown and will require further characterization of transplant and blood donors to elucidate.

We hypothesized that c-aAb-mediated functional inhibition of cytokines might manifest as altered profiles and trajectories of inflammatory, immunoregulatory and injury-related biomarkers and blood cells, and therefore found the extensive biomarker characterization of the present HSCT patient cohort to be an ideal way of exploring this. We found that several high-titre c-aAbs were associated with a range of biomarkers and blood cells at baseline, suggesting inherently differing immune profiles within these individuals prior to transplantation. Furthermore, we found associations between high-titre c-aAbs and biomarker trajectories over the course of HSCT, in which case patterns could be broadly divided into two different scenarios: either the biomarker remained consistently higher or lower relative to non-high titre individuals over the duration of the study, or the biomarkers’ path ultimately diverged at day 28, the end of follow-up. These scenarios indicate that inherently high titres of c-aAbs in patients might be associated with differences in key immunological signalling molecules both pre- and post-HSCT in patients. The finding that high-titre GM-CSF c-aAbs were associated with increased monocytes pre-HSCT and neutrophil counts post-HSCT was somewhat surprising. However, the functional roles of GM-CSF have recently been re-evaluated to be less related to promoting cell growth and more related to mediating inflammation^28,29^. When investigating the patient cases with the highest observed titres of the 6 c-aAbs pre-HSCT, many biomarkers and blood cell counts were again substantially elevated or reduced compared to the cohort average, indicating that these cases had a differing immune profile compared to the rest of the cohort.

Although the HSCT procedure has drastically improved over time through developments in conditioning regimens and donor matching^30^, adverse effects, such as GvHD, are still present. Cytokines are key factors in this condition; several proinflammatory cytokines, including IL-4, IL-6 and IL-17, serve as markers of chronic GvHD, and many current treatments for GvHD involve targeting cytokines^31^. Based on their immunomodulatory potential and a prior study of kidney-transplanted patients, which found that high-titre IL-10 c-aAbs were associated with increased odds of cardiac disease^32^, we therefore hypothesized that high-titre c-aAbs may influence clinical HSCT outcomes. We however found no evidence for this. Together, this suggests that the potential immunomodulatory effect of the c-aAbs observed in this cohort was insufficient to substantially affect these outcomes or was a result of limited power, scope and follow-up of this study. As five out of six of the highest titre c-aAb cases developed GvHD, this also indicates that the relatively small-sized cohort was unable to capture the full influence of the very highest-titre c-aAb.

In terms of the limitations and perspectives of the study, the results may be affected by both type I and type II errors: The modest size, limited follow-up and heterogeneity of the cohort (type II error) and multiple testing due to the number of c-aAb and biomarker/blood cell analyses (type I error). The present study is however exploratory, and our findings warrants confirmation and further investigation in larger cohorts with longer follow-up time, which would also allow for greater adjustment of potential confounders, such as age, sex, and malignancy of disease, and for investigation of the cause of the observed differences in biomarker concentrations and blood cell counts. Additional knowledge of c-aAb status in blood and especially transplant donors would also be highly relevant for proper interpretation. The strengths of the study include the extensive characterization of the cohort, both in terms of clinical variables and biomarker measurements, and it is the first study of c-aAbs in the context of HSCT.

In conclusion, this first study of cytokine-specific autoantibodies in HSCT demonstrates the presence of c-aAbs in patients undergoing HSCT, with prevalences resembling those healthy individuals. Furthermore, the c-aAb levels displayed a high degree of stability over time, including at high titres, and few patients appeared to “seroconvert” and become c-aAb high-titre positive during the course of HSCT. Furthermore, high-titre c-aAbs against GM-CSF and IL-10 in particular were associated with altered biomarker concentrations and blood cell counts pre-HSCT, as well as altered trajectories of several biomarkers over the course of HSCT. Our study suggests that high-titre c-aAbs may persist in HSCT patients and thus may exert immunomodulatory functions pre- and post-HSCT, as suggested by associations with a range of HSCT relevant measurements, including blood cell counts and cytokine concentrations.

## Methods

### Patients

The cohort comprised 131 adult patients who received a first HSCT with myeloablative conditioning at Rigshospitalet, Copenhagen (Copenhagen University Hospital), in the period from June 2015 to August 2018. Clinical data were registered prospectively in a local database, with the end of follow-up on December 1^st^, 2018. Table 1 shows the patient and transplant characteristics of the 131 included patients. The median age at HSCT was 49.6 years, and 46% were female. All transplants were allogeneic, and most patients were transplanted for a malignant diagnosis (91%) with an allograft of peripheral blood stem cells (65%) from an HLA-matched unrelated donor (66%). All patients provided informed written consent regarding the usage of their materials for research. The study was performed in accordance with the Helsinki Declaration and was approved by the Greater Copenhagen Regional Ethics Committee (approval nr. H-18012746).

### Transplant procedures

As GvHD prophylaxis, most patients received a calcineurin inhibitor (cyclosporin or tacrolimus) in combination with short-course methotrexate. Unless GvHD was present, cyclosporin was stopped at day + 180, whereas tacrolimus was tapered from day + 56 until day + 180. Additionally, anti-thymocyte globulin was added to the conditioning regimen in 1) mismatched unrelated donor transplants, 2) transplants for acute leukaemia when peripheral blood was the allograft source, and the conditioning regimen was not fludarabine and treosulfan, and 3) transplants for nonmalignant diseases. For infection prophylaxis, all patients were isolated in HEPA-filtered single bedrooms from initiation of their conditioning regimen until the absolute neutrophil count (ANC) was ≥ 0.5E9 cells/L in peripheral blood. When ANC was < 0.5E9 cells/L, patients received i.v. ceftazidime. Moreover, all patients received trimethroprim-sulfamethoxazole and valacyclovir from engraftment until the end of immunosuppressive therapy and oral fluconazole from day 0 to + 75. No patients received GM-CSF during HSCT.

### Plasma samples

The 131 patients had a total of 389 EDTA plasma samples available in the Centre of Excellence for Personalized Medicine of Infectious Complications in Immune Deficiency (PERSIMUNE) Research Biobank at Rigshospitalet, Copenhagen (www.persimune.dk). Of the 389 samples, 123 were collected pre-HSCT before the start of conditioning (between days − 72 and -12), 92 were collected around day + 7 (between days 0 and + 0), 78 were collected around day + 14 (between days + 11 and day + 24), and 96 were collected around day + 28 (between days + 25 and + 37). Samples were collected by peripheral venous puncture and centrifuged at 1800 × *g* at 5 °C for 10 min; the separated plasma was then aliquoted and stored at − 80 °C in the research biobank.

### Cytokine autoantibody measurements

Cytokine-specific autoantibodies (c-aAbs) against GM-CSF, IFNα, IFNγ, IL-1α, IL-6 and IL-10 were measured in duplicate for all plasma samples, as previously described^33^. Patients were classified as having high or nonhigh levels of the respective c-aAbs, with high levels defined as having an average c-aAb median fluorescence intensity (MFI) above the 95th percentile of the average cohort c-aAb MFI. The threshold was set to the 95th percentile due to the limited number of patients in the cohort to obtain a sufficient group size for analyses.

The stability of c-aAb levels over time was approximated by calculating coefficients of variation (CV%) for each patient for all measured c-aAb signals over time as the mean c-aAb signal/standard deviation (SD) * 100. Mann–Whitney *U*-tests were used to compare CVs between patients with high and non-high c-aAb levels. “Seroconversions” for c-aAb signals were defined as a factor of 5 or greater difference between the highest c-aAb signal and the lowest, provided the highest signal was above the 90th percentile.

### Biomarker analyses

A panel of 26 biomarkers was measured in all plasma samples. Concentrations of HMGB-1, IL-1β, IL-4, IL-6, IL-7, IL-8, IL-10, IL-12p70, IL-17-A, nucleosomes, Reg3A, ST2, TGF-β1 and TNFα were measured using ELISA kits (all from R&D Systems, Minneapolis, MN, except for HMGB-1 [MyBioSource, San Diego, CA], IL-4 [Abcam, Cambridge, UK] and nucleosomes [Roche, Indianapolis, IN]), and concentrations of CD40L, gp130, IFN-γ, IL-2Rα, IL-6Rα, IL-15, IL-22, IL-23, E-selectin, syndecan-1, thrombomodulin and TNFRI were measured using customized, magnetic bead-based, multi- or monoplex assays (R&D Systems). The magnetic beads were analysed on a Luminex LX-200 instrument (R&D Systems).

### Clinical outcomes

Engraftment was defined as the first of three consecutive days after HSCT with the patient achieving an ANC > 0.5E9 cells/L in peripheral blood. Acute GvHD was diagnosed and graded by the Keystone criteria^34^, and chronic GvHD was diagnosed by the treating physician based on clinical symptoms^35^. Infections were defined as a positive bacterial or mycobacterial culture from peripheral blood, as defined previously^36^, bronchoalveolar lavage fluid, ascites fluid or tracheal aspirate.

### Standard haematology

Information on leukocyte and platelet counts during HSCT was obtained from the PERSIMUNE Data Warehouse, which contains data on all routine biochemical analyses performed in patients during HSCT. To align cell counts with the biomarker data models and promote an even distribution of measurements, cell counts performed closest to days 0, + 7, + 14, and + 28 were selected within the following ranges: Day 0 if date < 4 days post-HSCT, day + 7 if date >  = 4 & < 11 days post-HSCT, day + 14 if date >  = 11 & < 17 days post-HSCT, and day + 28 if date >  = 26 & date <  = last day of c-aAb measurement for the patient.

### Statistical analyses

Pre-HSCT patient (age, sex, malignancy of disease, Karnofsky score, smoking status, BMI, CD34 cell count, conditioning regimen) and donor (age, match, stem cell source) characteristics (n = 123) were compared according to both continuous c-aAb signals and dichotomized c-aAb levels with high-titre vs. nonhigh-titre levels, applying the threshold levels displayed in Table 2 based on the 95th percentiles of c-aAb MFI. Continuous c-aAb signals were associated with continuous variables by Spearman correlation and compared with binary or categorial variables using two-tailed Mann–Whitney *U*-tests or Kruskal–Wallis tests depending on whether there were two or more outcomes. Analysis of continuous variables, including biomarker concentration and blood cell count, in patients with high-titre vs. non-high-titre c-aAbs pre-HSCT was performed using the Mann–Whitney *U*-test, and analyses of high titre c-aAb association with binary and categorical variables were performed using Fisher’s exact test. Spearman correlation, Mann–Whitney *U*-tests and Kruskal–Wallis tests were all chosen due to the nonparametric distributions of the variables.

Differences in biomarker concentrations and blood cell counts pre-HSCT (n = 123) and at day 28 (n = 96) were also tested by Mann–Whitney *U*-tests for “seroconverting” individuals expressing increasing c-aAbs over time versus individuals who did not. There were too few cases of decreasing c-aAbs over time to perform statistical analyses for this scenario. The occurrence of significant overlap between c-aAb signals was analysed by pairwise Spearman correlations for continuous c-aAb signals and Fisher’s exact test for high-titre levels of c-aAb.

To reveal the influence of the c-aAb levels on biomarker- and blood cell trajectories before and after HSCT, a linear repeated measures mixed-effects model including group (high-titre c-aAbs), time (0, 7, 14, 28 days) and group × time interaction with a subject-specific random intercept was constructed (n = 131). Biomarkers and blood cells were used as dependent variables and were log-transformed in the event of nonnormally distributed residuals. Whether variables were raw or transformed, only models with normally distributed residuals were considered valid and reported. The significance of group, time, and group × time effects were assessed in post hoc likelihood ratio tests. If the group or group × time interaction was statistically significant, subsequent mixed-effects models assessed the statistical significance of high-titre c-aAbs at individual time points by including a group × time interaction term as a predictor. Models were not reported if the group × time interactions alone were statistically significant, and values for the high-titre c-aAb group oscillated on days 7 and 14, as these scenarios were interpreted as biologically irrelevant fluctuations. The mixed model analyses were conducted with c-aAbs and time assumed to be independent of each other because prior studies have found a high stability of c-aAbs^3^.

Finally, we investigated the influence of c-aAb levels on select clinical outcomes, including engraftment, death, acute GvHD and infection (n = 131) by applying logistic and Cox proportional hazards regression models with high-titre c-aAbs as an independent variable, and clinical outcomes as dependent variables. Cox models for analysis with infection as an outcome were limited to 1 year of follow-up after transplant, and all cox were censored in the event of death or end of follow-up.

Due to the limited size of the cohort, we refrained from performing multivariate testing.

Data analyses were conducted using Stata (StataCorp. 2019. *Stata Statistical Software: Release 16*. College Station, TX: StataCorp LLC.). *P*-values < 0.05 were considered statistically significant.

## Supplementary Information

Supplementary Information 1.

## Data Availability

Data are not available for other researchers and belong to the patients and researchers attached to this particular study.

## References

[1] Bousfiha A (2020). Human inborn errors of immunity: 2019 update of the IUIS phenotypical classification. J. Clin. Immunol..

[2] von Stemann JH (2017). Prevalence and correlation of cytokine-specific autoantibodies with epidemiological factors and C-reactive protein in 8,972 healthy individuals: Results from the Danish blood donor study. PLoS ONE.

[3] Galle P, Svenson M, Bendtzen K, Hansen MB (2004). High levels of neutralizing IL-6 autoantibodies in 0.1% of apparently healthy blood donors. Eur. J. Immunol..

[4] von Stemann JH (2020). Cytokine autoantibodies are associated with infection risk and self-perceived health: Results from the Danish blood donor study. J. Clin. Immunol..

[5] Nanki T (2013). Suppression of elevations in serum C reactive protein levels by anti-IL-6 autoantibodies in two patients with severe bacterial infections. Ann. Rheum. Dis..

[6] Puel A (2008). Recurrent staphylococcal cellulitis and subcutaneous abscesses in a child with autoantibodies against IL-6. J. Immunol..

[7] Bloomfield M (2019). Anti-IL6 autoantibodies in an infant with CRP-less septic shock. Front. Immunol..

[8] Harada M (2021). Subcutaneous injection of interferon gamma therapy could be useful for anti-IFN-γ autoantibody associated disseminated nontuberculous mycobacterial infection. J. Infect. Chemother..

[9] Krisnawati DI (2019). Functional neutralization of anti-IFN-γ autoantibody in patients with nontuberculous mycobacteria infection. Sci. Rep..

[10] Bastard P (2020). Autoantibodies against type I IFNs in patients with life-threatening COVID-19. Science.

[11] Troya J (2021). Neutralizing autoantibodies to type I IFNs in >10% of patients with severe COVID-19 pneumonia hospitalized in Madrid, Spain. J. Clin. Immunol..

[12] Walter JE (2015). Broad-spectrum antibodies against self-antigens and cytokines in RAG deficiency. J. Clin. Invest..

[13] Kisand K (2008). Interferon autoantibodies associated with AIRE deficiency decrease the expression of IFN-stimulated genes. Blood.

[14] Meager A (2006). Anti-interferon autoantibodies in autoimmune polyendocrinopathy syndrome type 1. PLoS Med..

[15] Meyer S (2016). AIRE-deficient patients harbor unique high-affinity disease-ameliorating autoantibodies. Cell.

[16] Ku CL, Chi CY, von Bernuth H, Doffinger R (2020). Autoantibodies against cytokines: Phenocopies of primary immunodeficiencies?. Hum. Genet..

[17] Ben-Dov I, Segel MJ (2014). Autoimmune pulmonary alveolar proteinosis: Clinical course and diagnostic criteria. Autoimmun. Rev.

[18] Kitamura T (1999). Idiopathic pulmonary alveolar proteinosis as an autoimmune disease with neutralizing antibody against granulocyte/macrophage colony-stimulating factor. J. Exp. Med..

[19] Toullec L (2020). High levels of anti-GM-CSF antibodies in deep infiltrating endometriosis. Reprod. Sci..

[20] Bonneau J (2015). Systematic review: New serological markers (anti-glycan, anti-GP2, anti-GM-CSF Ab) in the prediction of IBD patient outcomes. Autoimmun. Rev..

[21] Perrineau S, Guery R, Monnier D, Puel A, Lanternier F (2020). Anti-GM-CSF autoantibodies and Cryptococcus neoformans var. grubii CNS vasculitis. J. Clin. Immunol..

[22] Saijo T (2014). Anti-granulocyte-macrophage colony-stimulating factor autoantibodies are a risk factor for central nervous system infection by *Cryptococcus gattii* in otherwise immunocompetent patients. MBio.

[23] Viola GM (2021). Disseminated cryptococcosis and anti-granulocyte-macrophage colony-stimulating factor autoantibodies: An underappreciated association. Mycoses.

[24] Jouvenne P, Fossiez F, Banchereau J, Miossec P (1997). High levels of neutralizing autoantibodies against IL-1 alpha are associated with a better prognosis in chronic polyarthritis: A follow-up study. Scand. J. Immunol.

[25] Lee JH (2000). Anti-A isoagglutinin as a risk factor for the development of pure red cell aplasia after major ABO-incompatible allogeneic bone marrow transplantation. Bone Marrow Transpl..

[26] Stussi G (2009). Prevention of pure red cell aplasia after major or bidirectional ABO blood group incompatible hematopoietic stem cell transplantation by pretransplant reduction of host anti-donor isoagglutinins. Haematologica.

[27] Tovey MG, Lallemand C (2011). Immunogenicity and other problems associated with the use of biopharmaceuticals. Ther. Adv. Drug Saf..

[28] Becher B, Tugues S, Greter M (2016). GM-CSF: From growth factor to central mediator of tissue inflammation. Immunity.

[29] Hamilton JA (2019). GM-CSF-dependent inflammatory pathways. Front. Immunol..

[30] Juric MK (2016). Milestones of hematopoietic stem cell transplantation—From first human studies to current developments. Front. Immunol..

[31] Zeiser R, Blazar BR (2017). Acute graft-versus-host disease—Biologic process, prevention, and therapy. N. Engl. J. Med..

[32] Lund KP (2019). IL-10-specific autoantibodies predict major adverse cardiovascular events in kidney transplanted patients—A retrospective cohort study. Transpl. Int..

[33] Guldager DK (2015). A rapid, accurate and robust particle-based assay for the simultaneous screening of plasma samples for the presence of five different anti-cytokine autoantibodies. J. Immunol. Methods.

[34] Przepiorka D (1995). 1994 Consensus conference on acute GVHD grading. Bone Marrow Transpl..

[35] Lee SJ, Vogelsang G, Flowers ME (2003). Chronic graft-versus-host disease. Biol. Blood Marrow Transpl..

[36] Gjaerde LI, Moser C, Sengeløv H (2017). Epidemiology of bloodstream infections after myeloablative and non-myeloablative allogeneic hematopoietic stem cell transplantation: A single-center cohort study. Transpl. Infect. Dis..

