# Supplementary material for: Cytokine autoantibodies are stable throughout the haematopoietic stem cell transplantation course and are associated with distinct biomarker and blood cell profiles
Source: Sci Rep. 2021 Dec 14;11:23971. doi: 10.1038/s41598-021-01952-6 (PMC8671426; doi:10.1038/s41598-021-01952-6)
Supplement: Supplementary file 1 — Supplementary Information 1. [file 41598_2021_1952_MOESM1_ESM.docx]

|  | Table S1: Overlap of c-aAb signals -average MFI | | | | | | | |
| --- | --- | --- | --- | --- | --- | --- | --- | --- |
|  | Test | Day | GM-CSF c-aAbs | IL-10 c-aAbs | IFNα c-aAbs | IFNy c-aAbs | IL-1α  c-aAbs | IL-6  c-aAbs |
| IL-10  c-aAbs | Continuous c-aAbs * | 0 | **0.56**  **P < 0.0001** |  |  |  |  |  |
|  |  | +28 | **0.53**  **P < 0.0001** |  |  |  |  |  |
|  | c-aAb High titer ** | Average | **2**  **P = 0.045** |  |  |  |  |  |
| IFNα  c-aAbs | Continuous c-aAbs * | 0 | **0.49**  **P < 0.0001** | **0.65**  **P < 0.0001** |  |  |  |  |
|  |  | +28 | **0.47**  **P < 0.0001** | **0.58**  **P < 0.0001** |  |  |  |  |
|  | c-aAb High titer ** | Average | 1  P = 0.325 | 1  P = 0.325 |  |  |  |  |
| IFNγ  c-aAbs | Continuous c-aAbs * | 0 | **0.35**  **P < 0.0001** | **0.54**  **P < 0.0001** | **0.39**  **P < 0.0001** |  |  |  |
|  |  | +28 | **0.42**  **P < 0.0001** | **0.52**  **P < 0.0001** | **0.43**  **P < 0.0001** |  |  |  |
|  | c-aAb High titer ** | Average | 0 | 0 | 0 |  |  |  |
| IL-1α  c-aAbs | Continuous c-aAbs * | 0 | **0.33**  **P = 0.0002** | **0.51**  **P < 0.0001** | **0.46**  **P < 0.0001** | **0.57**  **P < 0.0001** |  |  |
|  |  | +28 | **0.28**  **P = 0.0062** | **0.40**  **P = 0.0001** | **0.34**  **P = 0.0009** | **0.34**  **P = 0.0009** |  |  |
|  | c-aAb High titer ** | Average | 1  P = 0.325 | 1  P = 0.325 | **2**  **P = 0.045** | 1  P = 0.325 |  |  |
| IL-6  c-aAbs | Continuous c-aAbs * | 0 | **0.43**  **P < 0.0001** | **0.46**  **P < 0.0001** | **0.46**  **P < 0.0001** | **0.52**  **P < 0.0001** | **0.33**  **P = 0.0001** |  |
|  |  | +28 | **0.35**  **P = 0.0005** | **0.43**  **P < 0.0001** | **0.43**  **P < 0.0001** | **0.42**  **P < 0.0001** | 0.19  P = 0.0681 |  |
|  | Cc-aAb High titer ** | Average | 0 | 0 | 0 | **2**  **P = 0.045** | 1  P = 0.325 |  |

- *Spearman correlation for c-aAbs: Rho and p-value reported
- **Fisher’s exact test for c-aAb high positive groups: N overlap and p-value reported. High titer defined as > 95^th^ percentile MFI cut-off for average c-aAb/person

| Table S2: High-titer c-aAbs vs cells and biomarkers, pre-HSCT* | | | |
| --- | --- | --- | --- |
| Biomarker/cell | Cohort median (IQR) | High-titer** median (IQR) | P-value*** |
|  | GM-CSF c-aAbs | | |
| Monocytes (10E9 cells/L) | 0.9 (0.6; 1.3) | 0.2 (0; 0.5) | 0.015 |
|  | IL-10 c-aAbs | | |
| IL-7 (pg/ml) | 3.1 (1.5; 5.7) | 0.9 (0.6; 1.3) | 0.005 |
|  | IFNα c-aAbs | | |
| IL-6 (pg/ml) | 2.6 (1.5; 5.0) | 7.1 (3.3; 10.6) | 0.049 |
| IL-17A (pg/ml) | 0.1 (0.01; 0.3) | 0.3 (0.2; 1.3) | 0.019 |
| TNFα (pg/ml) | 1.3 (0.9; 1.8) | 2.1 (1.3; 2.9) | 0.030 |
| ST2 (pg/ml) | 19,555 (14,642; 25,609) | 28,856 (24,557; 31,527) | 0.003 |
|  | IFNγ c-aAbs | | |
| Syndecan-1 (pg/ml) | 2,243 (1,920; 2,700) | 1,766 (1,691; 2,096) | 0.018 |
|  | IL-1α c-aAbs | | |
| ST2 (pg/ml) | 27,415 (19,769; 33,144) | 19,647 (14,642; 25,628) | 0.048 |
| Lymphocytes (10E9 cells/L) | 0.1 (0; 0.2) | 0.2(0.1; 0.4) | 0.008 |
|  | IL-6 c-aAbs | | |
| Thrombomodulin (pg/ml) | 6,423 (5,228; 8,006) | 4,823 (3,341; 5,755) | 0.004 |

* Significant associations shown.

** High titer defined as > 95^th^ percentile MFI cut-off for average c-aAb/person

*** Mann-Whitney *U*-test, concentration or cell count comparison between c-aAb high/non-high titer groups, p-value reported

| Table S3: Case descriptions for highest pre-HSCT c-aAb levels | | | | | | | | | |
| --- | --- | --- | --- | --- | --- | --- | --- | --- | --- |
|  | Cohort median (Q1; Q3) | Cohort  10-90 percentile | GM-CSF c-aAb Case (MFI = 8,016) | IL-10 c-aAb Case (MFI = 8,010) | IFNα c-aAb Case (MFI = 17,567) | IFNγ c-aAb Case (MFI = 15,246) | IL-1α c-aAb Case (MFI = 24,614) | IL-6 c-aAb Case (MFI = 19,906) | Multiple c-aAbs positive (not IL-6 nor IFNγ) |
| Age at HSCT (years) | 50 (35.5; 58.7) | 22.8; 64.6 | 70.9↑↑ | 59.5↑ | 61.9↑ | 65.1↑↑ | 56 | 35.5↓ | 51 |
| Disease | See Table 1 | | CML | MDS | AML | AML | MDS | Diamond-Blackfan anaemia | MDS |
| Death | No (71.0 %)  Yes (29.0 %) | | No | Yes | Yes | No | No | No | No |
| Take | No (6.5 %)  Yes (93.6 %) | | Yes | Yes | Yes | Yes | Yes | Yes | Yes |
| aGVHD | No (58.1 %)  Yes (41.9 %) | | No | No | Yes | Yes | Yes | Yes | No |
| cGVHD | No (56.5 %)  Yes (43.6 %) | | Yes | Yes | No | Yes | Yes | Yes | No |
| Relapse | No (80.2 %)  Yes (19.9 %) | | No | No | Yes | No | No | No | No |
| SecondTX | No (97.0 %)  Yes (3.1 %) | | No | No | No | No | No | No | No |
| TNFα (pg/ml) | 1.3 (0.9; 1.9) | 0.7; 2.5 | 2.3↑ | 1.2 | 1.8 | 0.5 ↓↓ | 0.8↓ | 1.0 | 2.4↑ |
| IL-1b (pg/ml) | 0.1 (0.04; 0.2) | 0.004; 0.3 | 0.1 | 2.0 ↑↑ | 0.3↑↑ | 0.01↓ | 0↓↓ | 0.1 | 0.02↓ |
| IL-4 (pg/ml) | 1.6 (1.0; 3.3) | 0.5; 6.1 | 3.1 | 3.1 | 2.6 | 1.5↓ | 3.4↑ | 0.3 ↓↓ | 0.2↓↓ |
| IL-6 (pg/ml) | 2.7 (1.5; 5.9) | 1.0; 9.0 | 3.4 | 1.3↓ | 8.8↑ | 4 | 2.4 | 2.3 | 17.0↑↑ |
| IL-7 (pg/ml) | 3.0 (1.3; 5.6) | 0.5; 10.4 | 2.5 | 1.2↓ | 2.8 | 0.2 ↓↓ | 2.5 | 2.6 | 0.8↓ |
| IL-8 (pg/ml) | 8 (5; 15) | 3; 26 | 11 | 160 ↑↑ | 18↑ | 10 | 17↑ | 5 | 149↑↑ |
| IL-10 (pg/ml) | 1.2 (0.9; 1.8) | 0.5; 3.0 | 2.1↑ | 2.9↑ | 0.6↓ | 1.4 | 1.6 | 1.8 | 1.8 |
| IL-12p70 (pg/ml) | 0.03 (0; 0.4) | 0; 0.7 | 0.3 | 0.5↑ | 0 | 0 | 0.5 | 0 | 0 |
| IL-15 (pg/ml) | 4.7 (2.7; 9.0) | 2.0; 20.4 | 6.6 | 4.5 | 12.9↑ | 6.3 | 5.7 | 2.4↓ | 6.8 |
| IL-17A (pg/ml) | 0.1 (0.01; 0.3) | 0; 0.7 | 0.7 ↑↑ | 0↓↓ | 0.2 | 0.7↑↑ | 0.2 | 0.1 | 6.4↑↑ |
| IL-22 (pg/ml) | 0.5 (0.2; 0.8) | 0; 1.4 | 0 | 1.5↑↑ | 0.7 | 0↓↓ | 0.4 | 1.2↑ | 3.9↑↑ |
| IL- 23 (pg/ml) | 0 (0; 0) | 0; 10.8 | 0 | 0 | 59.9↑↑ | 0 | 0 | 0 | 0 |
| gp130 (pg/ml) | 383,144 (330,034; 441,233) | 296,525; 493,914 | 281,095 ↓↓ | 347,388 | 316,900↓ | 456,443↑ | 323,631↓ | 292,855↓↓ | 403,240 |
| IFNγ (pg/ml) | 0 (0; 0) | 0; 5.2 | 0 | 0 | 0 | 0 | 0 | 0 | 6.5↑↑ |
| sTNFRI (pg/ml) | 1,793 (1,486; 2,291) | 1,222; 3,089 | 2,280 | 1,685 | 2,830↑ | 1,313↓ | 1,045↓↓ | 1,278↓ | 2,310↑ |
| IL-2Ra (pg/ml) | 2,776 (2,202; 3,632.) | 1,594; 4,588 | 3,325 | 4,721↑↑ | 2,611 | 2,272 | 2,595 | 2,790 | 4,196↑ |
| sIL-6R (pg/ml) | 66,310 (54,807; 83,818) | 47,298; 99,704 | 112,254↑↑ | 39,964↓↓ | 78,414 | 48,906↓ | 47,298 ↓↓ | 41,005 ↓↓ | 47,576↓ |
| TGFβ1 (pg/ml) | 31.7 (17.8; 48.3) | 12.8; 67.4 | 19.9 | 66.2↑ | 39.1 | 29.1 | 14.3↓ | 39.1 | 18.3 |
| REG3a (pg/ml) | 7,485 (5,197; 11,021) | 4,028; 19,582 | 7,992 | 7,498 | 8,807 | 5,193↓ | 19,545↑ | 4,337↓ | 22,095↑↑ |
| ST2 (pg/ml) | 20,134 (15,079; 26,194) | 11,741; 34,335 | 23,293 | 16,986 | 24,557 | 22,240 | 21,363 | 11,350↓↓ | 44,617 |
| Thrombomodulin (pg/ml) | 6,190 (5,209; 7,968) | 4,277; 9,052 | 7,054 | 5,250 | 5,494 | 5,754 | 5,908 | 4,822↓ | 8,479↑ |
| Syndecan-1 (pg/ml) | 2,228 (1,902; 2,684) | 1,691; 3,230 | 3,565 ↑↑ | 2,584 | 2,349 | 1,562↓↓ | 2,513 | 2,226 | 2,990↑ |
| E-selectin (pg/ml) | 21,611 (15,433; 26,253) | 10,814; 35,694 | 24,083 | 19,782 | 14,149↓↓ | 19,500 | 2,849↓↓ | 25,024 | 8,317↓↓ |
| HMGB1 (pg/ml) | 816 (669; 1,142) | 552; 1,747 | 526↓↓ | 791 | 990 | 880 | 564↓ | 913 | 539↓↓ |
| Nucleosomes (enrichment factor) | 6.6 (4.1; 13.4) | 2; 32.7 | 2.6↓ | 1.1↓↓ | 5.1 | 8.6 | 5.8 | 1.7↓↓ | 5 |
| CD40 L (pg/ml) | 1,585 (559; 3,649) | 195; 5,595 | 3,233 | 3,057 | 2,680 | 330↓ | 2,167 | 1,891 | 187↓↓ |
| Basophils (10E9 cells/L) | 0 (0; 0.01) | 0; 0.02 | 0.01 | 0 | 0.01 | 0 | 0.01 | 0 | 0.01 |
| Eosinophils (10E9 cells/L) | 0.02 (0; 0.1) | 0; 0.1 | 0 | 0 | 0 | 0.01 | 0.01 | 0 | 0 |
| Leukocytes (10E9 cells/L) | 0.4 (0.1; 1.1) | 0.1; 1.9 | 3 ↑↑ | 0.1 | 0.2 | 0.6 | 1.6 | 0.2 | 0.10 |
| Neutrophils (10E9 cells/L) | 0.4 (0.1; 1) | 0.1; 2.2 | 2.3 ↑↑ | 0.2 | 0.5 | 0.5 | 0.4 | 0.3 | 0.1↓↓ |
| Lymphocytes (10E9 cells/L) | 0.1 (0; 0.2) | 0; 0.4 | 0.1 | 0.2 | 0.5↑↑ | 0 | 0.4↑↑ | 0.3↑ | 0.10 |
| Monocytes (10E9 cells/L) | 0 (0; 0.1) | 0; 0.3 | 0.4 ↑↑ | 0 | 0.4↑↑ | 0 | 0 | 0 | 0.6↑↑ |
| Platelets (10E9 cells/L) | 62 (17; 111) | 9; 143 | 10↓ | 115↑ | 107 | 13↓ | 118 | 44 | 4↓↓ |

*Median, 10^th^ and 90^th^ percentile reported. ↑↑ indicates cases with concentrations ≥90^th^ percentile, ↓↓ indicates concentrations ≤10^th^ percentile. ↑ indicates cases with concentrations ≥75^th^ percentile, and ↓ indicates concentrations ≤25^th^ percentile. Elevated concentrations are highlighted in red; reduced concentrations are highlighted in blue.


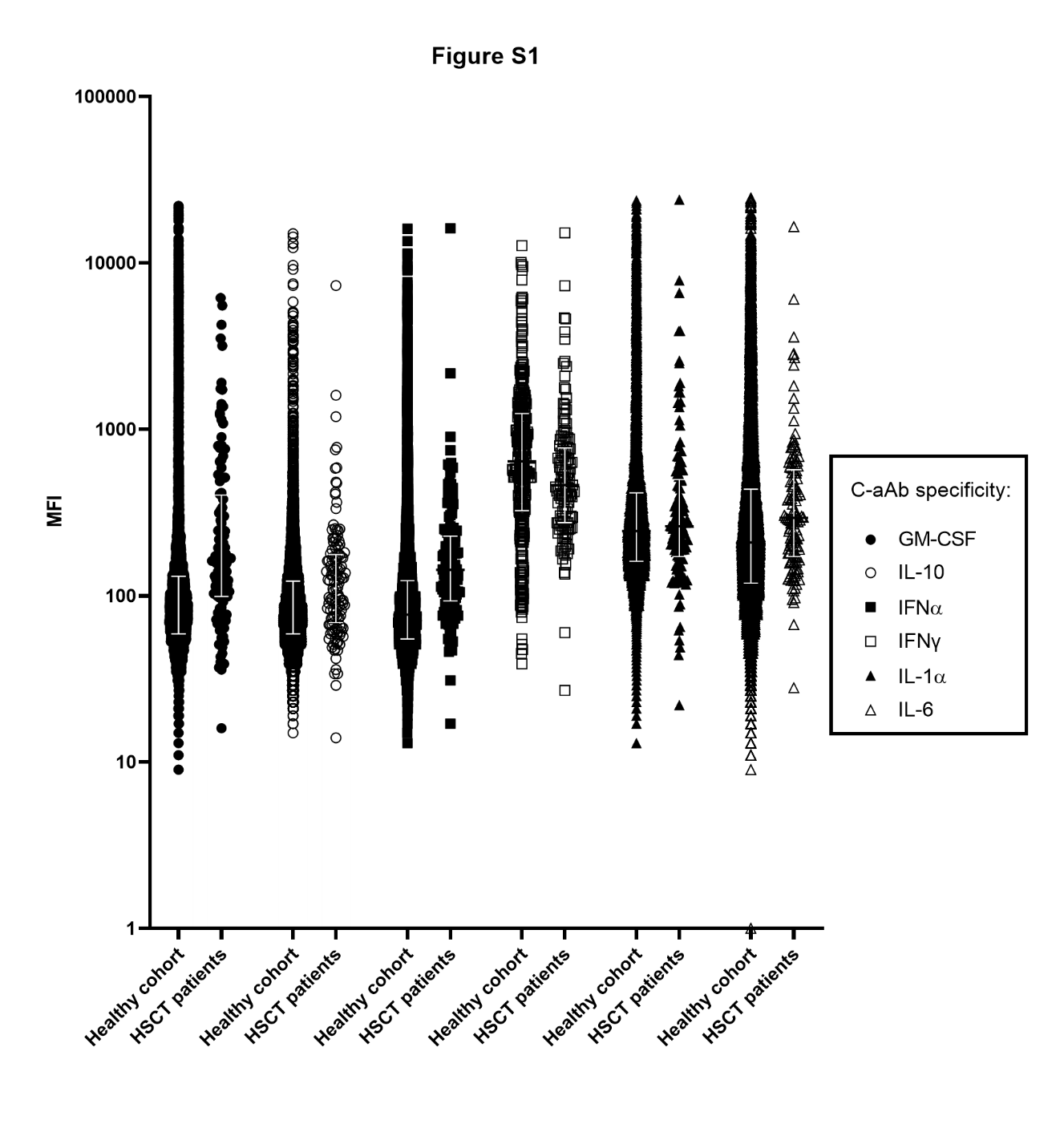


|  | GM-CSF c-aAbs | | IL-10 c-aAbs | | IFNα c-aAbs | | IFNy c-aAbs | | IL-1a c-aAbs | | IL-6 c-aAbs | |
| --- | --- | --- | --- | --- | --- | --- | --- | --- | --- | --- | --- | --- |
| Cohort | Healthy | HSCT | Healthy | HSCT | Healthy | HSCT | Healthy | HSCT | Healthy | HSCT | Healthy | HSCT |
| Median (IQR) | 85 (59; 131) | 160 (99; 402) | 81 (59; 121) | 111 (69; 176) | 77 (55; 123) | 142 (93; 227) | 641 (326; 1240) | 462 (275; 774) | 245 (161; 415) | 262 (171; 499) | 209 (119; 437) | 293 (172; 572) |
| 95^th^ percentile | 585 | 1,143 | 293 | 582 | 421 | 605 | 3,971 | 2,567 | 2,509 | 2,499 | 2,552 | 2,431 |
| 99^th^ percentile | 5,487 | 5,551 | 1,051 | 1,604 | 2,129 | 2,173 | 9,384 | 7,301 | 9,001 | 7,866 | 7,461 | 6,064 |

Fig. S1: c-aAb in healthy individuals vs HSCT patient comparison

Plot of the distribution of c-aAb MFI signals in the present HSCT cohort (n = 131) and in previously screened healthy individuals (n = 8.972 for GM-CSF, IL-10, IFNα, IL-1α and IL-6 c-aAbs, and n = 330 for IFNy c-aAbs).

For the HSCT patients, average c-aAb values across all measurements were used. White bars indicate medians with IQRs. The attached table contains medians with IQRs as well as 95^th^ and 99^th^ percentile cut-offs for MFI in the healthy and patient groups.
